# Insuffizienzfrakturen der Wirbelsäule in Abhängigkeit von der spongiösen Knochendichte: Eine in-vitro-Studie

**DOI:** 10.1007/s00132-022-04261-6

**Published:** 2022-05-23

**Authors:** Guido Schröder, Dirk Flachsmeyer, Claus Maximilian Kullen, Julian Ramin Andresen, Marko Schulze, Laura Hiepe, Hans-Christof Schober, Reimer Andresen

**Affiliations:** 1 Klinik für Orthopädie und Unfallchirurgie, Warnow Klinik Bützow, Am Forsthof 3, 18246 Bützow, Deutschland; 2 Institut für Diagnostische und Interventionelle Radiologie/Neuroradiologie, Westküstenklinikum Heide, Akademisches Lehrkrankenhaus der Universitäten Kiel, Lübeck und Hamburg, Heide, Deutschland; 3 Medizinische Fakultät, Sigmund-Freud-Privatuniversität, Wien, Österreich; 4 Institut für Anatomie und Zellbiologie, Universität Bielefeld, Bielefeld, Deutschland; 5 Institut für Anatomie, Universitätsmedizin Rostock, Rostock, Deutschland; 6 Klinikum Südstadt Rostock, Klinik für Innere Medizin IV, Akademisches Lehrkrankenhaus der Universität Rostock, Rostock, Deutschland

**Keywords:** Knochenmineralgehalt, Computertomographie, Leiche, Osteoporose, Wirbelkörper, Bone mineral density, Computed tomography, Corpse, Osteoporosis, Vertebral body

## Abstract

**Hintergrund:**

Das Risiko für osteoporotische Insuffizienzfrakturen (Fx) am Achsenskelett steigt mit zunehmender Abnahme der Knochendichte, wobei sich thorakal und thorakolumbal eine Häufung findet. Um die unterschiedliche Verteilung von Fx entlang der Wirbelsäule (WS) besser zu verstehen, wurden morphologische und osteodensitometrische Untersuchungen mittels Computertomographie (CT) in den verschiedenen WS-Abschnitten durchgeführt. Zudem war zu klären, ob die bei CT-Untersuchungen aus anderen Indikationen gefunden Hounsfield-Einheiten (HE) mit der Knochendichte korrelieren und Anlass für eine osteologische Diagnostik sein könnten.

**Material und Methoden:**

Von 26 Körperspenden wurden die gesamten WS in einem Plexiglas-Wasser-Phantom fixiert und mittels hochauflösende Spiral-CT analysiert. Zusätzlich erfolgte die Messung der CT-morphologischen Spongiosadichte in HE von C3 bis S2 (624 Wirbelkörper). Der Knochenmineralgehalt (KMG, mg/ml) wurde ermittelt und zur Abschätzung einer Osteoporose (OPO) herangezogen.

**Ergebnisse:**

Bei allen WS lag eine OPO vor. Bei einem KMG unterhalb von 60 mg/ml fanden sich signifikant vermehrte Sinterungsfrakturen im thorakalen und thorakolumbalen Bereich. Osteoporotische Insuffizienzfrakturen im HWS-Bereich fanden sich insgesamt nicht. Die Spongiosadichte war signifikant höher in den zervikalen (Median 188,6 HE) als in den lumbalen (Median 63,6 HE) und sakralen (Median 25,5 HE) Wirbelkörpern aller untersuchten WS.

**Schlussfolgerung:**

Ein KMG-Verlust der Wirbelkörperspongiosa führt zu einem erhöhten Fx-Risiko, welches sich auch bei den verwendeten WS findet. Jedoch wird im zervikalen Bereich ein scheinbarer Schwellenwert für das Auftreten von Sinterungsfrakturen nicht unterschritten. Einen Schwellenwert für HE zu finden, wäre für die klinische Praxis relevant.

## Abstract (en)

**Background:**

The risk of osteoporotic insufficiency fractures (Fx) at the axial skeleton increases with decreasing bone density, with an accumulation in the thoracic and thoracolumbar regions. To better understand the differential distribution of Fx along the spine, morphological and osteodensitometric studies were performed by computed tomography (CT) in the various spine sections. In addition, we aimed to clarify whether Hounsfield units (HU) found on CT examinations from other indications correlate with bone density and could be grounds for osteologic diagnosis.

**Material and methods:**

The entire spines of 26 body donors were fixed in a Plexiglas water phantom and analyzed by high-resolution spiral CT. In addition, CT morphological cancellous bone density was measured in HU from C3 to S2 (624 vertebral bodies). Bone mineral density (BMD, mg/ml) was calculated and used to estimate osteoporosis (OPO).

**Results:**

OPO was present in all spines. Significantly increased sintering fractures were found in the thoracic and thoracolumbar regions when BMD was below 60 mg/ml. Fx in the cervical spine area were not found overall. Cancellous bone density was significantly higher in the cervical (median 188.6 HU) than in the lumbar (median 63.6 HU) and sacral (median 25.5 HU) spine.

**Discussion:**

BMD loss of vertebral body cancellous bone leads to an increased risk of Fx, which is also found in the cadaver spines. However, an apparent threshold for the occurrence of sintering fractures is not undercut in the cervical region. Finding a threshold for HU would be relevant to clinical practice.

Die OPO stellt für die betroffenen Patienten bei Auftreten von Frakturen ein ernstes Problem dar. In Deutschland sind circa 8 Mio. Menschen von OPO betroffen [22]. Die Inzidenz für klinisch auffällige osteoporotische Wirbelkörperfrakturen beträgt circa 1,4 Mio. weltweit [23]. Aufgrund fehlender Diagnostik wird eine OPO erst spät oder nicht erkannt [24]. Insbesondere bei älteren Personen werden eine Vielzahl von CT-Untersuchungen durchgeführt. Eine routinemäßige Ermittlung der Hounsfield-Einheiten des Knochens im Rahmen dieser Untersuchungen könnte hier eine Lücke schließen.

## Hintergrund und Fragestellung

Die OPO zählen zu den metabolischen Knochenerkrankungen, bei denen durch eine Verminderung der Knochenmasse, -struktur und -funktion eine Fraktur bereits bei einem Niedrigenergietrauma eintreten kann [32]. Osteoporotische Frakturen finden sich unabhängig von Geschlecht und Alter vor allem im Bereich des distalen Radius, des proximalen Femurs und der Wirbelsäule (WS) [21]. Wirbelkörperfrakturen gehen mit gesundheitlichen und wirtschaftlichen Belastungen einher, erhöhen die Morbidität und Mortalität und beeinträchtigen die Lebensqualität [30]. Osteoporotische Wirbelkörperfrakturen treten bei deutlich reduzierter spongiöser Knochendichte vor allem thorakal, thorakolumbal und sakral – nicht jedoch zervikal auf [37, 38]. Eine höhere Knochenmasse und eine größere Interkonnektivität der Spongiosa im Bereich der Halswirbelsäule (HWS) wurde in einer früheren histomorphometrischen Arbeit beschrieben [20]. Da Wirbelkörperfrakturen häufig klinisch stumm verlaufen, kommt der Bildgebung nicht nur nach dem Auftreten von Symptomen, sondern auch beim Screening eine wichtige Rolle zu. Die Knochendichte als wichtiger Faktor der Festigkeit wird mit verschiedenen Methoden ermittelt, einfach wäre die Angabe der Hounsfield Einheiten (HE). Insbesondere im Hinblick auf eine präventive Behandlung einer osteoporotischen Fraktur, aber auch im Rahmen einer optimalen chirurgischen Vorbereitung eines Wirbelsäuleneingriffs, ist die Bestimmung der Knochenqualität entscheidend für den Behandlungserfolg [10]. Als Goldstandard für die Bestimmung der Knochendichte und Identifizierung einer Osteoporose gilt die Dual-Energie-Röntgenabsorptiometrie (DEXA) [17].

Eine Bewertung der HE in der Standard-CT könnte eine Schätzung der Knochendichte liefern, die diagnostische Leistung verbessern und unnötige Strahlenbelastung reduzieren [10].

Untersuchungen zu HE in der gesamten Wirbelsäule sind selten. Die Ermittlung der HE in den WS-Abschnitten und eine Einordnung in den Knochenverlust der Wirbelsäule mit dem sich entwickelnden Frakturrisiko ist von Interesse. Inwieweit Messungen an anderen Orten als der Lendenwirbelsäule (LWS) eine Rolle spielen, ist für die klinische Praxis (CT HWS, CT Thorax) relevant [14]. Um die Verteilung der HE über die gesamte WS zu verstehen und zusätzliche Informationen zur Knochenqualität zu generieren, wurden mittels CT morphologische und osteodensitometrische Untersuchungen in den verschiedenen Wirbelsäulenabschnitten von 26 Körperspenden durchgeführt.

## Methoden

### Studiendesign und Ethik

Die nachfolgende multizentrische, klinische, In-vitro-Untersuchung wurde durch die zuständige regionale Ethikkommission der Universitätsmedizin geprüft und genehmigt (Nr. A 2017–0072). Alle Probanden waren Teilnehmer des Körperspendeprogramms der Universitätsmedizin und hatten zu Lebzeiten die freiwillige Einwilligung erteilt, ihren Körper nach dem Tod der wissenschaftlichen Forschung zur Verfügung zu stellen. Die Methoden, die zur Gewinnung von menschlichem Gewebe genutzt wurden, entsprechen den ethischen Standards der Deklaration von Helsinki. Die anamnestischen Angaben beschränkten sich auf die Diagnosen der Todesbescheinigung. Die Gruppenzuweisung erfolgte anhand der Lage der Wirbelkörper in den einzelnen Wirbelsäulenabschnitten (HWS, BWS, LWS, SWS).

### Ein- und Ausschlusskriterien

Die Einschlusskriterien der klinischen Untersuchung waren das Vorliegen von 24 Wirbelkörpern pro entnommene Wirbelsäule und das fortgeschrittene Lebensalter. Ausschlusskriterien waren relevante anatomische Deformitäten, das Vorliegen einer Wachstumsretardierung, schwere Knochenerkrankungen, wie Tumoren, Knochenmetastasen, Morbus Paget, Wirbelfusionen, Skoliose oder die Bildung von Blockwirbeln sowie vorangegangene Operationen mit Fremdmaterial in der Wirbelsäule.

### Bildgebende Diagnostik

**Figure 1:**
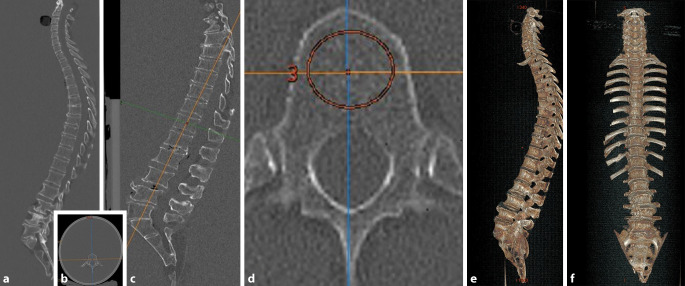

Von 26 Körperspenden wurden die gesamten Wirbelsäulen, zur Simulation eines homogenen, anatomisch analogen Körperumfangs, möglichst luftfrei, in ein Plexiglas-Wasser-Phantom (KG-Rohr aus Hart-Polyvinylchlorid, PVC-U) mit einem Durchmesser von 25 cm und einer Länge von 125 cm fixiert (Abb. 1**a** und **b**, [2]). Danach wurde ein hochauflösendes Spiral-CT (GE Revolution EVO/64 Zeilen CT/laterales Scanogramm, axiale Schichtdicke < 1 mm, sowie axiale und sagittale Reformation mit einer Schichtdicke von 2 mm) durchgeführt. In den sagittal reformierten Schnittbildern (Abb. 1c) erfolgte die Detektion und Gradeinteilung von Wirbelkörperdeformitäten [19] durch zwei unabhängige Radiologen. Zur Visualisierung der gesamten Wirbelsäulenanatomie erfolgte eine 3‑D-Volumendarstellung an einer externen Workstation (GE AW-Server ® Version 2.0. Vermessung der Wirbelsäulen in GE CentricityRIS‑i ® Version 5.0, GE Healthcare, Solingen, Deutschland) (Abb. 1e).

### Bestimmung der Hounsfield-Einheiten und der Knochendichte

Die Messung der CT-morphologischen Spongiosadichte in HE der einzelnen Wirbelköper von HWK 3 bis SWK2 (insgesamt 624 Wirbelkörper) erfolgte jeweils durch eine manuell positionierte Region von Interesse im spongiösen Raum. Die Region von Interesse wurde so groß wie möglich gewählt, wobei der Wirbelkörperkortex ausgespart wurde (Abb. 1d).

Für die weitere Berechnung verwendeten wir den Mittelwert des Wirbelkörpers in der axialen, koronaren und sagittalen Ebene. Aus diesen drei Werten wurde der Mittelwert der HE des jeweiligen Wirbelkörpers berechnet. Um Intraobserver-Unterschiede zu vermeiden, wurde die HE-Messung in unserer Studie immer von demselben Radiologen durchgeführt.

Die Bestimmung des spongiösen Knochenmineralgehalts erfolgte rechnerisch anhand der Formel: QCT-Wert = 17,8 + 0,7 × HE [10]. Der Mittelwert angegeben in mg/ml wurde zur Abschätzung einer Osteoporose herangezogen.

Darüber hinaus wurde die Knochenmineralgehaltsbestimmung mithilfe der quantitativen CT (GE Revolution EVO/64 Zeilen Computertomograph sowie Mindways Software 3D Volumetric QCT Spine, Austin, Tx, USA) vorgenommen. Diese erfolgte im Volumenblock auf Höhe von LWK 1, LWK 2 und LWK 3. Der Mittelwert – angegeben in mg/ml – wurde zur Einordnung der errechneten Knochendichte herangezogen.

### Statistik

Die erhobenen Daten wurden mit dem statistischen Softwarepaket SPSS, Version 23.0 (SPSS 10 Inc., Chicago, Il, USA) analysiert. Die Beschreibung der quantitativen Merkmale erfolgte bei parametrischen Tests jeweils als Mittelwert (MW), Standardabweichung (SD) und Anzahl (*n*) der verfügbaren Beobachtungen, sie wurden mithilfe des Intervalls Mittelwert ± Standardabweichung (M ± SD) dargestellt. Bei nichtparametrischen Tests erfolgte die Darstellung jeweils als Median mit dazugehörigem erstem und drittem Quartil (Q1–Q3). Für Vergleiche zwischen den Gruppen kam der Kruskal-Wallis-Test zum Einsatz. Die Wahl zwischen diesen wurde in Abhängigkeit vom Resultat des Shapiro-Wilk-Tests auf Normalverteilung getroffen. Bei signifikanten Ergebnissen führten die Autoren paarweise Vergleiche durch. Gleichzeitig wurden die Effektstärken nach Cohen (d) berechnet und Werte < 0,5 als kleiner, zwischen 0,5 und 0,8 als mittlerer sowie > 0,8 als großer Effekt angenommen. Alle *p*-Werte sind das Resultat zweiseitiger statistischer Tests: prinzipiell wird *p* < 0,05 als signifikant angesehen.

## Ergebnisse

Insgesamt wurden Daten von 624 Wirbelkörpern aus 26 humanen Körperspenden ausgewertet, darunter waren 9 Männer und 17 Frauen im Alter von 66–102 Jahren (Durchschnittsalter 81,3 ± 8,0 Jahre). Vier Wirbelsäulen wurden aufgrund von Metastasen, einer fortgeschrittenen Skoliose, einer idiopathischen skelettalen Hyperostose und einer Blockwirbelbildung von der weiteren Untersuchung ausgeschlossen. Der Body-Mass-Index (BMI) der Gesamtgruppe betrug im Mittel 22,0 ± 5,2 kg/m^2^. Die verfügbare Krankengeschichte beschränkte sich auf die Todesursache. Einen anamnestischen Überblick bietet die Tab. 1.

**Table 1:** 

Parameter	Gesamtgruppe (*n* = 26)	Männer (*n* = 9)	Frauen (*n* = 17)
Alter (Jahren)	81,3 ± 8,0	78,4 ± 6,3	82,8 ± 8,5
Geschlecht (Männer/Frauen)	9/17	9	17
Body-Mass-Index (kg/m^2^)	22,0 ± 5,2	23,5 ± 6,0	21,2 ± 4,7
Entnommene Segmente	C3-S2	C3-S2	C3-S2
Wirbelkörperfrakturen (MW±SD)	2,0 ± 1,3	2,0 ± 1,3	2,1 ± 1,3
Anzahl untersuchter Wirbelkörper (*n*)	624	216	408

Variablen ausgedrückt als Mittelwert±Standardabweichung (MW±SD) und Anzahl an Observationen (*n*)

**Figure 2:**
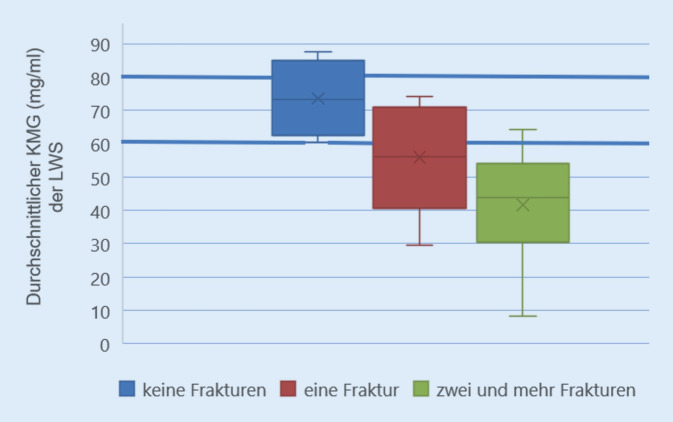

**Figure 3:**
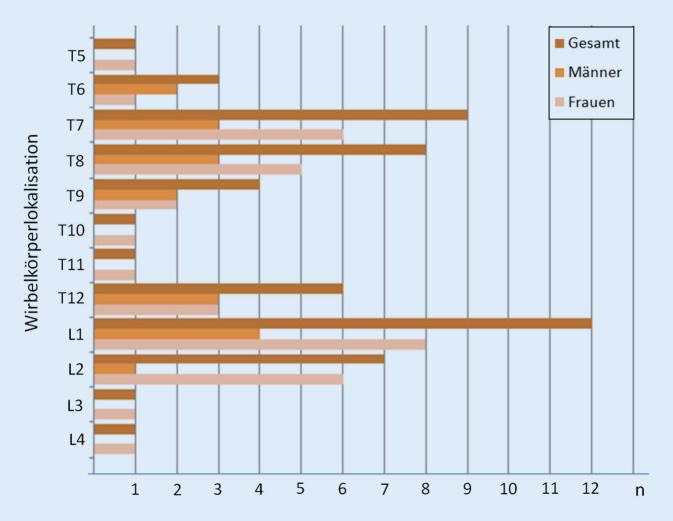

Bei allen Wirbelsäulen lag eine Osteoporose vor. Bei einem Knochenmineralgehalt unterhalb von 60 mg/ml fanden sich signifikant vermehrte Sinterungsfrakturen im thorakalen und thorakolumbalen Bereich (Abb. 2). Geschlechterübergreifend war der LWK 1 mit insgesamt 12 Frakturen am häufigsten betroffen. Danach folgten der BWK 7 mit neun und der BWK 8 mit acht Frakturen. Bei Frauen brach der LWK 1 mit neun nachgewiesenen Frakturen am häufigsten. Danach folgten der LWK 2 und der BWK 7 mit jeweils sechs stattgehabten Frakturen. Bei den untersuchten männlichen Wirbelsäulen war ebenfalls der LWK 1 mit vier detektierten Frakturen am häufigsten betroffen. Danach folgten der BWK 7, 8 und 12 mit jeweils drei Frakturen. Oberhalb von BWK 5, speziell im HWS-Bereich fanden sich keine Frakturen. Einen Überblick über die ermittelten Frakturen in den unterschiedlichen Wirbelsäulenabschnitten gibt die Abb. 3.

**Figure 4:**
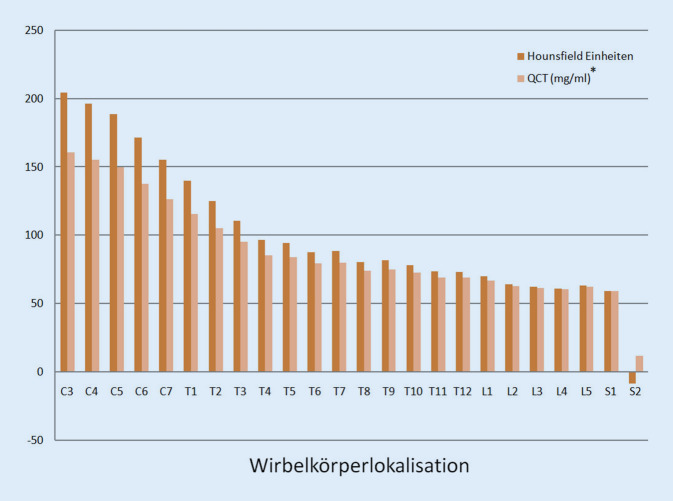

Darüber hinaus ermittelten wir die Spongiosadichte in HE. Die Spongiosadichte war signifikant (*p* < 0,001) höher in den zervikalen (188,58 HE im Median) als in den thorakalen (Median 88,17HE), lumbalen (Median 63,64 HE) und sakralen (Median 25,52 HE) Wirbelkörpern aller untersuchten Wirbelsäulen. Eine Verteilung der mittvertebralen Spongiosadichte über die gesamten Wirbelsäulen zeigt einen kontinuierlichen Dichteanstieg in Richtung der Halswirbelkörper (Abb. 4).

In einer Subgruppenanalyse wurden Männer und Frauen bezüglich der eingangs beschriebenen Parameter betrachtet. Unabhängig vom Geschlecht lagen in der HWS die höchsten HE vor (Tab. 2; Abb. 4). Hinsichtlich der ermittelten BMD zeigte sich eine signifikant höhere BMD in den Halswirbeln als in den Brust‑, Lenden- und Sakralwirbeln (Tab. 2; Abb. 4).

**Table 2:** 

–		Wirbelsäulenabschnitt							Gruppenvergleich ^*P*^			
Parameter	Gruppe	Gesamt Median (Q1–Q3)	HWS Median (Q1–Q3)	BWS Median (Q1–Q3)	LWS Median (Q1–Q3)	SWS Median (Q1–Q2)	HWS vs. BWS *p*-Wert (d)	HWS vs. LWS *p*-Wert (d)	HWS vs. SWS *p*-Wert (d)	BWS vs. LWS *p*-Wert (d)	BWS vs. SWS *p*-Wert (d)	LWS vs. SWS *p*-Wert (d)
Hounsfield-Einheiten	Gesamt	84,88 (65,48–136,26)	188,58 (163,48–200,52)	88,17 (78,67–107,09)	63,64 (61,58–66,96)	25,52 (−8,35–25,52)	0,024 (0,55)	< 0,001 (1,20)	0,001 (1,31)	0,024 (0,55)	0,026 (0,59)	0,554
	Männer	99,44 (82,22–153,67)	202,67 (181,72–216,67)	101,83 (91,08–119,0)	72,89 (71,06–82,22)	29,94 (−8,11–29,94)	0,024 (0,55)	< 0,001 (1,20)	0,001 (1,31)	0,024 (0,55)	0,026 (0,59)	0,554
	Frauen	77,74 (60,76–127,04)	173,18 (153,82–195,94)	80,53 (71,46–101,51)	56,71 (54,68–61,65)	23,18 (−8,47–23,18)	0,024 (0,55)	< 0,001 (1,22)	0,001 (1,28)	0,021 (0,56)	0,033 (0,57)	0,636
Knochenmineralgehalt (mg/ml)	Gesamt	77,22 (63,64–113,18)	149,80 (132,24–158,16)	79,52 (72,87–92,76)	62,22 (60,90–64,67)	35,66 (11,96–35,66)	0,024 (0,55)	< 0,001 (1,20)	0,001 (1,31)	0,024 (0,55)	0,026 (0,59)	0,554
	Männer	87,41 (75,36–125,37)	159,67 (145,01–169,47)	89,08 (81,56–101,10)	68,82 (67,54–75,36)	38,76 (12,12–38,76)	0,024 (0,55)	< 0,001 (1,20)	0,001 (1,31)	0,024 (0,55)	0,026 (0,59)	0,554
	Frauen	72,21 (60,34–106,73)	139,02 (125,48–154,96)	74,17 (67,82–88,86)	57,49 (56,07–60,75)	34,02 (11,87–34,02)	0,024 (0,55)	< 0,001 (1,22)	0,001 (1,28)	0,021 (0,56)	0,033 (0,57)	0,636

Die Ergebnisse sind für nicht normalverteilte Parameter als Median mit Angabe des 1. und 3. Quartils (Q1–Q3) dargestellt

*HWS* Halswirbelsäule, *BWS* Brustwirbelsäule, *LWS* Lendenwirbelsäule, *SWS* Sakralwirbelsäule, *d* Effektstärke, *P* paarweiser Vergleich

Die ermittelten HE korrelieren signifikant mit der errechneter BMD (r = 1,000, *p* < 0,001).

## Diskussion

Die vorliegende Untersuchung ermöglicht den Vergleich von spongiösem Knochen aus allen Abschnitten der Wirbelsäule von 26 Körperspenden im Alter von 66–102 Jahren. Der BMI war in der Gesamtgruppe niedrig normal, ein BMI geringer als 22 kg/m^2^ geht mit einer ansteigenden Frakturrate einher [27]. Zusammenhänge zwischen niedrigem Körpergewicht, niedrigem Fettanteil und einem höheren Frakturrisiko wurden mehrfach beschrieben [26, 27]. Alle untersuchten Probanden wiesen densitometrisch mit Knochenmineralgehaltswerten unter 80 mg/ml eine Osteoporose auf [18], wobei sich bei Werten unter 60 mg/ml obligat Sinterungsfrakturen fanden [3]. Der wichtigste Risikofaktor für diese Frakturen ist das hohe Alter der Patienten. Die Frakturraten steigen besonders im Alter über 70 Jahre [5]. Die Frakturen verteilten sich in typischer Weise auf die thorakolumbalen und lumbalen Abschnitte. Unterschiedliche Belastungen in den einzelnen Wirbelsäulenabschnitten spiegeln sich in der Frakturhäufigkeit wider. Die Frakturen traten vorrangig im mittleren Brustwirbelsäulenbereich (T7, T8) und im Übergangsbereich von Brustwirbelsäule zu Lendenwirbelsäule (T12 bis L1) auf [4, 15, 29]. Eine mögliche Erklärung für diese Frakturkaskade entlang der Wirbelsäule könnten die vorherrschenden Krümmungen der Wirbelsäule bieten [13]. So findet sich der Krümmungswendepunkt der Brustkyphose im mittleren Bereich der Brustwirbelsäule (T7, T8) wieder.

Es ist anzunehmen, dass lokale Veränderungen der Mikroarchitektur und Wirbelsäulenfunktion nachweisbar sind und eine Rolle spielen.

In der vorliegenden Untersuchung war der LWK 1 geschlechterunabhängig am häufigsten von einer Fraktur betroffen. Interessant ist, dass der LWK 1 mit 69,9 HE eine deutlich höhere Spongiosadichte aufweist als der LWK 4 mit 60,9 HE. Das lässt vermuten, dass neben der Spongiosadichte die oben genannten Faktoren eine wichtige Rolle bezüglich stattgehabter Frakturen spielen. Ermittlungen der HE von vollständigen Wirbelsäulen inklusive der Berechnung der dazugehörigen BMD sind bisher kaum erfolgt. Dabei stellen die HE einen normalisierten Index der Röntgenstrahlabschwächung basierend auf einer Skala von 1000 für Luft und 0 für Wasser dar. Der HE-Wert für Knochen liegt typischerweise zwischen 300 und 3000 [36]. Mithilfe der HE sind Aussagen über die Knochendichte möglich. Schreiber et al. [36] ermittelten eine signifikante Korrelation zwischen den T‑Werten der DEXA-Messung und den HE des gleichen Wirbelkörpers.

Choi et al. [12] sehen nach Abschluss ihrer Untersuchung neben der klaren Korrelation von HE und T‑Wert bei Patienten mit degenerativen Wirbelsäulenerkrankungen sogar einen Vorteil der HE-Bestimmung.

Die Messung der HE wäre einfach und damit eine opportunistische OPO-Diagnostik im Rahmen der häufigen CT-Untersuchungen.

DEXA-Messungen sind der Goldstandard in der OPO-Diagnostik und weltweit etabliert. Falsch hohe Werte können entstehen durch degenerative Veränderungen, Osteophyten, eine Osteochondrose, eine Skoliose und die vor der WS liegende eventuell verkalkte Aorta [31, 34]. Zudem können Positionierungsfehler zu Fehlinterpretationen führen [40]. Bei Verlaufsmessungen unter einer OPO-Therapie waren DEXA-Veränderungen an der Hüfte gesamt und am Schenkelhals prädiktiv für proximale Femur- und Wirbelkörperfrakturen. DEXA-Messungen an der Wirbelsäule waren nur für diesen Messort vorhersagend [8]. Eine differenzierte Betrachtung ist notwendig. Zumindest für die Wirbelkörper könnte die HE-Messung eine Option im klinischen Alltag darstellen.

Welche HE sich als potenzieller Grenzwert für den Nachweis von Osteoporose eignet, ist Gegenstand der Forschung. Schreiber et al. [36] erhoben für Patienten mit Osteoporose im Mittel 78,5 HE. Buckens et al. [9] kamen am LWK 1 auf einen Grenzwert von 99 HE. Allerdings muss kritisch hinterfragt werden, ob von der Messung nur eines Wirbelkörpers auf einen generalisierten Knochenabbau geschlossen werden kann. Dementsprechend sehen Scheyerer et al. [35] die Erhebung der HE an mindestens drei verschiedenen Lendenwirbelkörpern als wichtig an. Nach Auffassung der Autoren ist dabei weniger bedeutend, welcher LWK gemessen wird, sondern vielmehr, dass er frei von degenerativen (z. B. subchondrale Sklerosierung), posttraumatischen oder postoperativen Veränderungen ist. In der vorliegenden Untersuchung wurden für die Abschätzung einer Osteoporose die HE von LWK 1 bis LWK 3 gemessen. Im Mittel ergab sich für die Gesamtgruppe ein Wert von 65,4 HE. Schwaiger et al. [39] konnten mit ihrer Untersuchung zeigen, dass ab 63,8 HE verstärkt Frakturen auftreten. Das deckt sich mit den Ergebnissen unserer Studie. Zudem lagen die HE der LWS bei Frauen (57,9 HE im Mittel) deutlich unter denen der Männer (75,9 HE im Mittel), was die höhere Anzahl der Frakturen in dieser Gruppe erklären könnte.

Interessant sind die Daten einer jüngst publizierten Übersichtsarbeit zu HE-Cut-off-Werten im Rahmen der OPO-Diagnostik [1]. In Tab. 3 sind auszugsweise die Arbeiten von Berger-Groch et al. [6], Li et al. [28], Kim et al. [25], Pickhardt et al. [33] und Da Zou et al. [16] zusammengefasst. Ein Wert in der LWS von unter 136 HE sollte Beachtung finden. Zumeist wurde die LWS untersucht, auch Angaben zu HE-Werten der HWS liegen vor [14]. Eine vollständige Messreihe zu HE-Werten von C3 bis S1 wird von uns vorgelegt.

**Table 3:** 

Studiengruppe	Durchschnittsalter (Jahre)	HE-Cut-off-Werte						
		L1	L2	L3	L4	L5	S1	S2
Berger-Groch et al. [6]. 2020	72	–	–	–	< 62	< 58	< 68	–
Li et al. [28] 2018	67	< 136					–	–
Kim et al. [25] 2019	k. A.	≤ 95			–	–	–	–
Pickhardt et al. [33] 2013	59	135	–	–	–	–	–	–
Da Zou et al. [16] 2019	59	110	100	85	80	–	–	–

In der Untersuchung von Colantonio [14] wurden die HE-Werte der HWS mit DEXA-Messungen an der Hüfte verglichen und Grenzwerte für eine Osteoporose dargestellt. Dieser Aspekt ist von besonderem Interesse.

Hinsichtlich der Methodik der Messung der HE gab es bisher verschiedene Ansätze. Einige Autoren bestimmten die HE innerhalb des Wirbelkörpers in einer axialen Schicht im spongiösen Knochen [33]. Andere Autoren bestimmten HE in den sagittalen Schichten der CT-Untersuchung [39]. Unterschiede in der Validität bestanden dabei allerdings keine.

Auffällig ist in den Befunden insbesondere die signifikant höhere Knochendichte der Spongiosa der HWS im Vergleich zu der von BWS und LWS. Vergleichbare Ergebnisse sind in der Arbeit von Grote et al. [20] zu finden. Aufgrund der Ergebnisse ihrer histomorphometrischen Aufarbeitung der Knochenstruktur gehen sie davon aus, dass die Dichte des trabekulären Knochens in den Halswirbeln deutlich höher ist als im Bereich der Brust- und Lendenwirbel. Auch Schröder et al. [37] gelangten in ihrem Fallbericht sowie in einer ersten Pilotstudie [38] zu vergleichbaren Ergebnissen. In der vorliegenden Untersuchung unterschied sich die errechnete Knochendichte (BMD) in den einzelnen Wirbelsäulenabschnitten signifikant, wobei sich für die HWS die höchsten Werte ergaben. Grote et al. [20] zeigten mit ihrer Untersuchung, dass der Verlust an Knochenvolumen sogar im Altersgang in der HWS am geringsten ausfällt. So konnte bei den Halswirbeln 3 und 4 kein signifikanter altersbedingter Verlust der trabekulären Strukturdichte beobachtet werden. Dieser Befund deckt sich mit den Ergebnissen der vorliegenden Untersuchung.

Die Belastungsfähigkeit eines Wirbelsäulenabschnitts hängt von den Materialeigenschaften des Wirbelkörpers und von seinen geometrischen Abmessungen ab. Da die Materialeigenschaften von Knochengewebe im weitesten Sinne vorgegeben sind, können die Abmessungen der Wirbelkörper als Resultat eines Anpassungsvorgangs an die von außen und innen einwirkenden Lasten angesehen werden (Körpergewicht, Muskelaktivität, Vorspannung der Bandstrukturen, extern applizierte Kräfte) [11].

Welche klinische Relevanz die vorgestellten Ergebnisse aufweisen, sollte in Folgestudien untersucht werden, denn bisher fehlten sichere Kriterien für die Versorgung der osteoporotischen Wirbelkörperfraktur. Gängige Frakturklassifikationen ließen sich nicht oder nur eingeschränkt auf den älteren Menschen mit verminderter Knochenqualität und zahlreichen Komorbiditäten übertragen. Blattet et. al [7] empfehlen Augmentationstechniken mit Zement oder den Einsatz spezieller Schraubendesigns mit erhöhter Haltekraft im strukturschwachen Knochen. Dadurch vergrößert sich die Kontaktfläche zwischen Schraube und Knochen. Es lässt sich vor dem Hintergrund der vorgestellten Studienergebnisse vermuten, dass in Abhängigkeit von der Wirbelkörperlokalisation ein individuelles Schraubendesign vorteilhaft wirken kann.

## Limitationen

Bei der vorliegenden Studie handelt sich um eine vergleichende, deskriptive Untersuchung mit einer an das vorhandene Material gebundenen Fallanzahl. Komplexe statistische Verfahren konnten nur bedingt angewandt werden. Es wurden ausschließlich Körperspenden im höheren Lebensalter untersucht, Aussagen über die Knochenstruktur jüngerer Körperspenden sind daher nicht möglich. Gleichzeitig lagen kaum Angaben zu den jeweiligen Anamnesen der Spender vor, insbesondere zur Art und Dauer einer durchgeführten medikamentösen oder physikalischen Osteoporosebehandlung.

## Fazit für die Praxis

- Ein Verlust an Knochenmineralgehalt in der Wirbelkörperspongiosa führt zu einem erhöhten Frakturrisiko, welches sich auch bei den untersuchten Wirbelsäulen zeigte.
- Im zervikalen Bereich wird jedoch ein frakturkritischer Schwellenwert der spongiösen Knochendichte für das Auftreten von Sinterungsfrakturen, auch bei manifester Osteoporose, scheinbar nicht unterschritten.
- Bei klinischem Verdacht auf das Vorhandensein einer Osteopenie/Osteoporose können in CT-Untersuchungen von Thorax und Abdomen eine zusätzliche Dichtebestimmung (HE) im spongiösen Wirbelsäulenbereich weitere Hinweise liefern.

## Abkürzungen

BMD: Bone mineral density
BWK: Brustwirbelkörper
BWS: Brustwirbelsäule
COX: Cyclooxygenase
CT: Computertomographie
DEXA: Dual-Energie-Röntgenabsorptiometrie
Fx: Fraktur
GE: General Electric
HE: Hounsfield Einheiten
HWK: Halswirbelkörper
HWS: Halswirbelsäule
KG-Rohr: Kanalgrundrohr
KMG: Knochenmineralgehalt
LWK: Lendenwirbelkörper
LWS: Lendenwirbelsäule
MW ± SD: Mittelwert ± Standardabweichung
*n*: Anzahl
Nr: Nummer
OPO: Osteoporose
PVC: Polyvinylchlorid
Q1–Q3: Quartil 1 bis 3
QCT: quantitative Computertomografie
ROI: Range of Interest
SWK: Sakralwirbelkörper
SWS: Sakralwirbelsäule
WS: Wirbelsäuke
